# Future Time Perspective in the Work Context: A Systematic Review of Quantitative Studies

**DOI:** 10.3389/fpsyg.2017.00413

**Published:** 2017-03-28

**Authors:** Hélène Henry, Hannes Zacher, Donatienne Desmette

**Affiliations:** ^1^Psychological Sciences Research Institute, Université Catholique de LouvainLouvain-la-Neuve, Belgium; ^2^Institute of Psychology, University of LeipzigLeipzig, Germany; ^3^School of Management, Queensland University of Technology, BrisbaneQLD, Australia

**Keywords:** systematic review, future time perspective, FTP, socioemotional selectivity, lifespan

## Abstract

A core construct in the lifespan theory of socioemotional selectivity, future time perspective (FTP) refers to individuals’ perceptions of their remaining time in life. Its adaptation to the work context, occupational future time perspective (OFTP), entails workers’ perceptions of remaining time and opportunities in their careers. Over the past decade, several quantitative studies have investigated antecedents and consequences of general FTP and OFTP in the work context (i.e., FTP at work). We systematically review and critically discuss this literature on general FTP (*k* = 17 studies) and OFTP (*k* = 16 studies) and highlight implications for future research and practice. Results of our systematic review show that, in addition to its strong negative relationship with age, FTP at work is also associated with other individual (e.g., personality traits) and contextual variables (e.g., job characteristics). Moreover, FTP at work has been shown to mediate and moderate relationships of individual and contextual antecedents with occupational well-being, as well as motivational and behavioral outcomes. As a whole, findings suggest that FTP at work is an important variable in the field of work and aging, and that future research should improve the ways in which FTP at work is measured and results on FTP at work are reported.

## Introduction

For several decades, researchers have been investigating phenomena associated with temporal experience (Lewin, 1939; Wallace, 1956; Kastenbaum, 1961). For example, already Lewin (1939) suggested that people of all ages are influenced by their perceptions of the future. Since chronological age is strongly associated with the passage of time, Carstensen (1991) proposed that scholars should take perceptions of time into account when studying human development. Assuming that with age people become increasingly aware that their time is “running out,” Carstensen et al. (1999) define general *future time perspective (FTP)* as individuals’ perception of their remaining time in life. According to Carstensen’s socioemotional selectivity theory (e.g., Carstensen, 1991, 2006; Carstensen et al., 1999), the perception of time plays a fundamental role in the selection and pursuit of goals, in particular goals related to knowledge acquisition, social contact, and emotional experience. For example, the theory proposes that when time is perceived as limited, people emphasize positive emotional states and relationships with close social partners.

Over recent years, several studies have examined general FTP to improve understanding of associations between age and work outcomes (Rudolph, 2016). Seijts (1998) was the first to suggest investigating FTP in the work context. He argued that the future time span workers consider when making decisions will predict what type of goals they pursue and, consequently, their motivation and performance at work. Moreover, since the end of an individual’s career (i.e., retirement) is an important endpoint in life, older workers likely perceive their occupational future time as more limited than their younger colleagues (Stamov-Roßnagel, 2015). However, until 2009, no research had empirically examined antecedents and consequences of FTP in the work context. To address this gap in the literature, Zacher and Frese (2009) adapted FTP to the work context. They defined *occupational future time perspective (OFTP)* as workers’ perceptions of remaining time and opportunities in their careers.

Both general FTP and OFTP are examined in the work and organizational psychology literature. In this review article, we use the term “FTP at work” to refer to both general FTP and OFTP investigated in the work and employment context. So far, despite potential theoretical and empirical differences between general FTP (which refers to perceptions of remaining time and opportunities in life in general) and OFTP (which refers to perceptions of remaining time and opportunities in one’s career) no systematic review on these constructs exists. In addition, since 2009, several studies conducted in the work context have examined antecedents and/or consequences of either general FTP or OFTP. However, the differences between these constructs may limit comparisons of study results. Moreover, due to a lack of conceptual integration, there is currently no clear agenda for future research on FTP at work, and it is not possible to derive useful practical implications for managers and organizations. Given current changes in employment trends, such as the extension of the remaining time to work due to delayed retirement entry, we believe that it is timely and important to review and integrate the state of the knowledge on FTP at work.

We posit that this systematic review will contribute to the literature in several ways. First, we will distinguish studies that have measured general FTP in the work context and studies that have measured OFTP. Second, we will systematically review quantitative studies that have examined antecedents and/or consequences of FTP at work, and studies that have investigated its role as a mediator or as a moderator. Third, we will identify important conceptual and methodological issues that need to be addressed in future research, and we will outline practical recommendations.

## Theoretical Framework and Operationalization of FTP at Work

To clarify the two conceptualizations of FTP at work (i.e., general FTP and OFTP), this section aims to define both concepts in further detail before we present the methods and results of our systematic review.

### General Future Time Perspective

Early definitions of FTP characterized the construct as “a relatively general tendency to be concerned with future events” (Kastenbaum, 1961, p. 217) or as “the length of the future time span which is conceptualized” (Wallace, 1956, p. 240). Kastenbaum’s (1961) definition is closely related to the concept of *future orientation*, which refers to the relatively stable tendency of individuals to adopt a future temporal frame of mind when making decisions (Zimbardo and Boyd, 1999). Individuals with a strong future orientation tend to engage in future-oriented behaviors, such as planning and delaying gratification (Strathman et al., 1994; Qian et al., 2015). Future orientation has often been studied in relation to health and environmental behaviors (e.g., Strathman et al., 1994).

In contrast, Wallace’s (1956) definition is related to FTP as defined by Carstensen et al. (1999) in their socioemotional selectivity theory, that is, as individuals’ perceptions of their remaining time in life (see also Lang and Carstensen, 2002). According to socioemotional selectivity theory, goals change with age, such that older people prioritize emotionally meaningful goals and relationships with close social partners. By contrast, young people tend to prioritize instrumental goals, such as acquiring knowledge and extending their social networks. Socioemotional selectivity theory proposes that FTP explains these age-related changes in life goals. Empirical research has generally supported this assumption (e.g., Fung et al., 1999; Lang and Carstensen, 2002; Fung and Carstensen, 2004; Carstensen, 2006). FTP as defined by Carstensen et al. (1999) differs from temporal orientation constructs such as Zimbardo and Boyd’s (1999) future orientation (see also Shipp et al., 2009). While future orientation refers to rather stable modes of thought and behavior, FTP is a flexible and age-related construct that changes over time and across the lifespan (Cate and John, 2007). The reason for the malleability of FTP is that people become more and more aware that their time in life is running out when they grow older (Carstensen et al., 1999). Example items of Carstensen and Lang’s (1996) widely used general FTP scale are “Many opportunities await me in the future” or “Most of my life lies ahead of me” (see **Table 1**).

**Table 1:** General FTP and OFTP items.

Research focus	Items
General FTP (Carstensen and Lang, 1996)	(1) Many opportunities await me in the future
	(2) I expect that I will set many new goals in the future
	(3) My future is filled with possibilities
	(4) Most of my life lies ahead of me
	(5) My future seems infinite to me
	(6) I could do anything I want in the future
	(7) There is plenty of time left in my life to make new plans
	(8) I have the sense time is running out (reverse coded)
	(9) There are only limited possibilities in my future (reverse coded)
	(10) As I get older, I begin to experience time as limited (reverse coded)
OFTP (Zacher and Frese, 2009; Zacher, 2013)	(1) Many opportunities await me in my occupational future^∗^
	(2) I expect that I will set many new goals in my occupational future^∗^
	(3) My occupational future is filled with possibilities^∗^
	(4) I could do anything I want in my occupational future
	(5) There are only limited possibilities in my occupational future (reverse coded)
	(6) There is plenty of time left in my occupational life to make new plans
	(7) Most of my occupational life lies ahead of me^∗^
	(8) My occupational future seems infinite to me^∗^
	(9) I have the sense that my occupational time is running out (reverse coded)
	(10) As I get older, I begin to experience time in my occupational future as limited (reverse coded)^∗^

*^∗^Items that were included in the original OFTP scale by Zacher and Frese (2009)*.

Experimental studies have also shown that FTP is a malleable construct. For instance, Carstensen and Fredrickson (1998) found that young individuals of approximately the same age, but different in their health status (i.e., HIV negative, HIV positive without symptoms, and HIV positive with symptoms), preferred to spend time with close social partners when their chances of dying soon were higher (i.e., limited FTP). Moreover, FTP can be manipulated. For instance, Fung et al. (1999) induced a limited FTP among young and older participants by asking them to imagine that they will immigrate to another country in a few weeks; they also induced an open-ended FTP by asking participants to imagine that a new medial advance will allow them to live 20 more years than expected. They found that in the limited FTP condition, both young and older individuals preferred familiar social partners. In the open-ended FTP condition, older people’s preference for close social partners disappeared. Thus, age differences in the preference for close social partners may disappear when FTP is manipulated. Taken together, these findings suggest that not only individual factors, such as age, but also life circumstances may influence FTP.

According to Carstensen et al. (1999), FTP is a unidimensional and bipolar concept ranging from expansive to limited perceived time left. Challenging this notion, Cate and John (2007) argued that an aging person may perceive time as increasingly limited but not necessarily as less full of opportunities. Therefore, they suggested that FTP may be conceived in terms of a focus on opportunities (i.e., perceiving new goals and possibilities in one’s remaining lifetime) and as a focus on limitations (i.e., perceiving limitations and constraints in one’s remaining lifetime). In a series of cross-sectional and longitudinal studies, Cate and John (2007) provided evidence for this two-dimensional model of FTP. Other authors replicated this two-dimensional structure of FTP, and distinguished between limited (i.e., focus on limitations) and open-ended (i.e., focus on opportunities) FTP (Cozzolino et al., 2009; Rabinovich et al., 2010; Kooij and Van De Voorde, 2011; Kooij et al., 2013). Nevertheless, most studies on general FTP in the work context conceptualized FTP as a unidimensional construct (e.g., Bal et al., 2010; Baltes et al., 2014).

### Occupational Future Time Perspective

To adapt FTP to the work context, Zacher and Frese (2009) added the word “occupational” to each item of Carstensen and Lang’s (1996) general FTP scale (see **Table 1**). Example items are “Most of my occupational life lies ahead of me” (i.e., perceived remaining time at work) and “Many opportunities await me in my occupational future” (i.e., focus on opportunities at work). Therefore, OFTP refers to workers’ perceptions of remaining time and opportunities in their careers. Like general FTP, OFTP has been shown to change with age and over time. For instance, Weikamp and Göritz (2015) found in a six-wave study that OFTP decreases over time such that individuals perceived losses of remaining time and opportunities at work over 4 years. In particular, age appears to be more strongly negatively related to perceptions of remaining time at work, probably because most people retire within a defined age range (Zacher and Frese, 2009; Weikamp and Göritz, 2015). Age is less strongly associated with remaining opportunities at work, which suggest that this dimension of OFTP can be influenced by variables other than age, such as job characteristics (Zacher and Frese, 2009; Zacher et al., 2010).

Similar to Cate and John’s (2007) two-dimensional model of general FTP, Zacher and Frese (2009) distinguished two dimensions of OFTP: perceived remaining time at work (i.e., similar to the temporal dimension of general FTP, as defined by Carstensen et al., 1999) and focus on opportunities at work (i.e., similar to general focus on opportunities as defined by Cate and John, 2007). Several researchers have adopted this conceptualization in their studies (e.g., Weikamp and Göritz, 2015). However, similar to studies that investigated general FTP in the work context, studies that measured OFTP differ regarding the way they operationalize OFTP. For instance, some researchers choose to investigate only one dimension of OFTP, such as only focus on opportunities at work (e.g., Zacher et al., 2010; Schmitt et al., 2013b) or only remaining time at work (Kooij and Zacher, 2016); while others examined the two dimensions together (i.e., remaining time and focus on opportunities at work; e.g., Zacher and Frese, 2009; Weikamp and Göritz, 2015). Besides, in a later study, Zacher (2013) used a version of Carstensen and Lang’s (1996) FTP scale that was adapted to the work context and provided evidence for three distinct dimensions of OFTP: perceived remaining time, focus on opportunities, and focus on limitations.

## Method

### Inclusion/Exclusion Criteria

We set five inclusion/exclusion criteria before conducting our systematic review. First, since no research had empirically investigated FTP at work until Zacher and Frese (2009), we included only articles that were published between 2009, and December 2016. Second, we included only quantitative-empirical studies on antecedents and consequences of FTP at work and excluded review articles and articles using a qualitative approach. Third, we included only articles written in English language. Fourth, to distinguish studies on FTP at work from studies that investigated trait-like constructs, such as Shipp et al.’s (2009) future orientation, we selected only studies that measured FTP either with the original items from Carstensen and Lang (1996), or with the adapted items from Zacher and Frese (2009) or similar versions of their scale. Finally, we selected only studies on FTP in the work and employment context; articles that investigated general FTP outside the work domain were excluded. Therefore, we included only studies with samples of workers or job seekers (e.g., Zacher, 2013). Studies that sampled adolescents or students were not included.

### Literature Search

**FIGURE 1:**
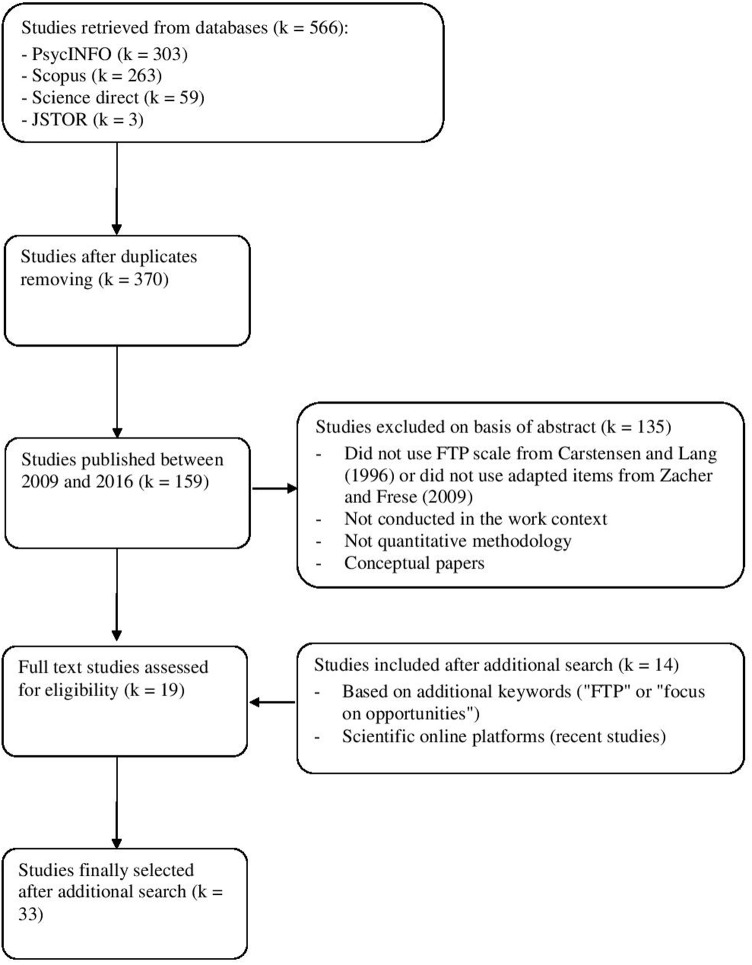
**Flow chart literature search process**.

We searched the electronic databases Scopus, PsycINFO, Science direct, and JSTOR, using the keyword “FTP.” We did not use keywords such as “focus on opportunities,” “focus on limitations,” or “remaining time” because these keywords identified studies that were not about FTP (for instance, when we used the keywords “remaining time” or “focus on limitations,” we found studies that included in their abstracts expressions such as “effects persisted over the remaining time” or “the discussion focuses on limitations”). We found more studies about FTP in the databases Scopus (*k* = 263) and PsycINFO (*k* = 303) than in the databases Sciencedirect (*k* = 59) and JSTOR (*k* = 3). This initial search resulted in 370 articles about FTP, after the removal of duplicates (see **Figure 1**).

In a second step, to select studies about FTP at work, we selected only articles published between 2009 and 2016. The number of studies identified in this step was 159. We then analyzed abstracts to select only quantitative-empirical studies on FTP that have been conducted in the work context, and that used the FTP scale by Carstensen and Lang (1996) or the items adapted by Zacher and Frese (2009). We excluded studies that used the Zimbardo Time Perspective Inventory (Zimbardo and Boyd, 1999; e.g., Anagnostopoulos and Griva, 2012) or the Consideration of Future Consequences Scale (e.g., Arnocky et al., 2014); studies that were conducted with student samples (e.g., Peetsma and van der Veen, 2011), children or adolescents (e.g., Duangpatra et al., 2009), very old adults outside of the work context (e.g., Kozik et al., 2015), and studies that used a qualitative methodology (e.g., Brown et al., 2012). We also excluded conceptual papers (e.g., Froehlich et al., 2016). This procedure resulted in 19 articles.

To ensure that we included all studies on FTP at work, we conducted additional searches in Scopus and Psycinfo with the keywords “FTP” and “focus on opportunities.” We found five additional studies that investigated only focus on opportunities at work (Zacher and Frese, 2009; Gielnik et al., 2012; Schmitt et al., 2013a,b; Zacher and Yang, 2016), which resulted in a total of 24 articles. Finally, to ensure that we did not omit the most recent articles on FTP at work (e.g., advance online publications) or articles published in journals that are not yet available in some databases (e.g., *Work, Aging, and Retirement*), we also searched for recent studies on FTP at work in scientific online platforms, such as ResearchGate and Academia.edu. This led us to find nine additional recent studies about FTP at work (e.g., Korff et al., 2016). In total, we found 33 articles that investigated general FTP (*k* = 17) or OFTP (*k* = 16) in the work context (see **Table 2**). Whenever longitudinal analyses were reported, we included relationships based from Time 1 to Time 2 data only. When the study included more than three measurement points, we calculated the average correlation coefficient (e.g., Weikamp and Göritz, 2015).

**Table 2:** Empirical studies on future time perspective (FTP) at work published over the past decade.

Authors and year	Sample^a^ and design	Measure and dimensions	Antecedents^b^	Consequences^b^
**General FTP at work**				
*Unidimensional operationalization*				
(1) Bal et al., 2013	Sample 1: 117 employees, mean age = 37 years, cross-sectional; Sample 2: 217 employees, mean age = 54.8 years, cross-sectional	FTP (10 items), general FTP	^∗^Age (-)	^∗^Continuance commitment (-)^∗^Normative commitment (-)
(2) Bal et al., 2010	176 post-retirement workers, 65–79 years, cross-sectional	FTP (10 items), general FTP		^∗^Employer developmental fulfillment (+)^∗^In-role obligations (-)^∗^Citizenship obligations (-) *High performance obligations (n.s.)*
(3) Baltes et al., 2014	104 older contracts workers, mean age = 69.20 years, three-wave study	FTP (10 items), general FTP	*Promotion focus (n.s.)*	^∗^Promotion focus (+)
(4) De Lange et al., 2011	90 employees, 22–61 years, two-wave study	FTP (seven items), general FTP	*/*	*Work motivation (n.s.)*
(5) Korff et al., 2016	913 employees, mean age = 41.9 years, cross-sectional	FTP (10 items), general FTP	^∗^HRM systems(^∗^Motivation enhancing practices [+]; *Knowledge, skills and abilities enhancing practices [n.s.]*;*Opportunity enhancing practices [n.s.])*	^∗^Job satisfaction (+)^∗^Affective organizational commitment (+)
(6) Oostrom et al., 2016	244 employees, 45–65 years, cross sectional	FTP (10 items), general FTP	^∗^Tasks and work responsibilities i-deals (+)	^∗^Employability (+)
(7) Park and Jung, 2015	555 employees, 18–57 years, cross sectional	FTP (10 items), general FTP	/	^∗^Occupational self-efficacy (+)^∗^Career commitment (+)^∗^Organizational commitment (+)
(8) Sia et al., 2015	234 female employees, 40–45 years, cross-sectional	FTP (10 items), general FTP	/	^∗^Physical, emotional, and cognitive work engagement (+)
(9) Treadway et al., 2010	291 managers, mean age = 30.6 years, cross-sectional	FTP (10 items), general FTP (and OFTP)	/	^∗^Career networking (+ for general FTP; *n.s. for OFTP*)^∗^Community networking (+ for general FTP; *n.s. for OFTP*)
(10) Treadway et al., 2011	291 employees, mean age = 30.6 years, cross-sectional, sample overlap with [9]	FTP (10 items), general FTP	/	^∗^Continuance commitment (-)*Affective commitment (n.s.)*
(11) Yeung et al., 2013	67 Chinese clerical employees, 19–58 years, 14-day experience sampling study	FTP (10 items), general FTP	/	^∗^Momentary task performance (+)
*Bidimensional operationalization*				
(12) Akkermans et al., 2016	186 taxi employees, mean age = 55.01 years, cross-sectional	Focus on opportunities (FO) (three items), remaining time (RT) (three items)		^∗^Intrinsic work motivation (+: RO and RT)^∗^Extrinsic work motivation (+ for RT; *n.s. for RO*)^∗^Motivation to continue working (+ for RT; *n.s. for RO*)
(13) Kooij et al., 2013	Study 1: 385 health care employees, mean age = 45.7 years, cross-sectional; Study 2: 1169 university employees, mean age = 42.5 years, sample overlap with [15]	FTP (five items), open-ended FTP, limited FTP	^∗^Age (- for open-ended FTP; + for limited FTP)	^∗^Growth motivations (+ for open-ended FTP; - for limited FTP [only in Sample 2])^∗^Esteem motivations (+ for open-ended FTP*; n.s. for limited FTP*) *Security motivations (n.s.) Generativity motivations (n.s.)*
(14) Kooij et al., 2016	287 university employees, mean age = 45.38 years, two-wave study, sample overlap with [13,15,17]	FTP (10 items), open-ended FTP, limited FTP	/	^∗^Job crafting (increasing job resources and challenging job demands; decreasing hindering JD) (+ for open-ended FTP; *n.s. for limited FTP*)
(15) Kooij and Van De Voorde, 2011	660 university employees, mean age = 43.9 ears, two-wave study	FTP (10 items), open-ended FTP, limited FTP	^∗^Subjective general health (+ for open-ended FTP; - for limited FTP)	^∗^Development motives (+ for open-ended FTP; - for limited FTP),^∗^Generativity motives (+ for limited FTP; *n.s. for open-ended FTP*)
(16) Zacher and de Lange, 2011	85 employees, mean age = 43.41 years, two-wave study	FTP (six items), focus on opportunities, focus on limitations	^∗^Age (- for focus on opportunities; *n.s. for focus on limitations*) ^∗^Promotion orientation (+ for focus on opportunities; *n.s. for focus on limitations*)^∗^Prevention orientation (+ for focus on limitations; *n.s. for focus on opportunities*)	*Promotion orientation (n.s.) Prevention orientation (n.s.)*
*Only remaining time*				
(17) Kooij et al., 2014	301 university employees, 19–67 years, four-wave study, sample overlap with [13,15]	FTP (four items), remaining time	^∗^Age (-)	^∗^Promotion focus (+)*Growth motives (n.s.)*
**Occupational FTP**				
*Unidimensional operationalization*				
(1) Bal et al., 2015	168 employees, 21–70 years, cross-sectional	OFTP (three items), overall OFTP (until retirement)	^∗^Age meta-stereotypes (-)*Negative age stereotypes (n.s.)*	^∗^Intention to retire (-)
(2) Ho and Yeung, 2016	199 Chinese clerical workers, 20–64 years, experimental study (scenarios)	OFTP (10 items), overall OFTP	/	^∗^Psychological distress (-)*Job stress (n.s.)*
*Bi(tri)dimensional operationalization*				
(3) Froehlich et al., 2016	282 employees, mean age = 41.85 years, cross-sectional	OFTP (seven items), focus on opportunities (FO), remaining time (RT)	^∗^Age (- for FO and RT)	^∗^Employability (^∗^Anticipation and optimization [+ for FO; *n.s. for RT*];^∗^Personal flexibility [+ for FO*; n.s. for RT*];*Occupational expertise [n.s.])*
(4) Kochoian et al., 2016	560 workers, 21–64 years, cross-sectional	OFTP (eight items), focus on opportunities (FO), constrained remaining time (RT)	^∗^Age (+ for constrained RT; - for FO)	^∗^Learning self-efficacy (+ for FO; - for constrained RT)^∗^Learning value (+ for FO; *n.s. for constrained RT*)
(5) Weikamp and Göritz, 2015	2180 workers, 18–65 years, six-wave study	OFTP (six items), focus on opportunities (FO), remaining time (RT)	^∗^Age (- for RT and RO)	/
(6) Weikamp and Göritz, 2016	312 workers, 21–64 years, three-wave study	OFTP (five items), focus on opportunities (FO), remaining time (RT)	/	^∗^Job satisfaction (+ for FO; *n.s. for RT*)^∗^OCBO > OCBI (+ for FO; *n.s. for RT*)
(7) Zacher, 2013	182 older job seekers, 43–77 years, cross-sectional	OFTP (10 items), focus on opportunities (FO), perceived remaining time (RT), focus on limitations	/	*^∗^Job search intensity*
(8) Zacher and Frese, 2009	176 workers, 19–60 years, cross-sectional	OFTP (six items), focus on opportunities (FO), remaining time (RT)	^∗^Age (- for FO; - for RT)^∗^Job complexity and job control (+ for FO; *n.s. for RT*)	/
*Only focus on opportunities*				
(9) Gielnik et al., 2012	84 business owners, 24–74 years, cross-sectional	OFTP (five items), focus on opportunities	^∗^Age (-)^∗^Mental health (+)	^∗^Venture growth (+)
(10) Gielnik et al., 2016	201 small business managers, 23–83 years, five-wave study	OFTP (five items), focus on opportunities	^∗^Age (-)	^∗^Business growth (+)
(11) Schmitt et al., 2013a	124 business owners, mean age = 52.7 years, two-wave study	OFTP (four items), focus on opportunities	^∗^General optimism (+)*Work engagement (n.s.)*	^∗^Work engagement (+)*General optimism (n.s.)*
(12) Schmitt et al., 2013b	Study 1: 174 employees of a manufacture, 16–64 years, cross-sectional; Study 2: 64 administrative employees, 20–62 years, daily diary study (5 days)	OFTP (five items), focus on opportunities	/	^∗^Work engagement (+)
(13) Zacher and Frese, 2011	133 employees, 16–65 years, cross-sectional	OFTP (four items), focus on opportunities	^∗^Age (-)^∗^Job complexity (+) ^∗^Use of SOC strategies (+)	/
(14) Zacher et al., 2010	168 employees, 19–64 years, cross-sectional	OFTP (three items), focus on opportunities	^∗^Age (-)^∗^Job complexity (+)	^∗^Work performance (+)
(15) Zacher and Yang, 2016	649 employees, 18–74 years, cross-sectional	OFTP (three items), focus on opportunities	^∗^Organizational climate for successful aging (+)	^∗^Job satisfaction (+)^∗^Organizational commitment (+)^∗^Motivation to continue working after official retirement age (+)
*Only remaining time*				
(16) Kooij and Zacher, 2016	Study 1: 175 employees, 19–69 years, cross-sectional; Study 2: 149 employees, mean age = 35.4 years, two-wave study	OFTP (three items), remaining time	^∗^Age (-)^∗^Work centrality (+)	^∗^Learning goal orientation (+)^∗^Attitude toward learning and development (+)

*FTP, Future time perspective (when items used refer to the future “in general”; Carstensen and Lang, 1996; Lang and Carstensen, 2002); OFTP, occupational future time perspective (when items used were adapted to the work context, Zacher and Frese, 2009); FO, focus on opportunities; RT, remaining time. ^a^Age range of the sample is provided whenever available; otherwise, mean age is reported. ^b^Antecedents/outcomes with an asterisk were significantly related to FTP at work; antecedents/outcomes in italics were not significantly related to FTP at work*.

## Results

**FIGURE 2:**
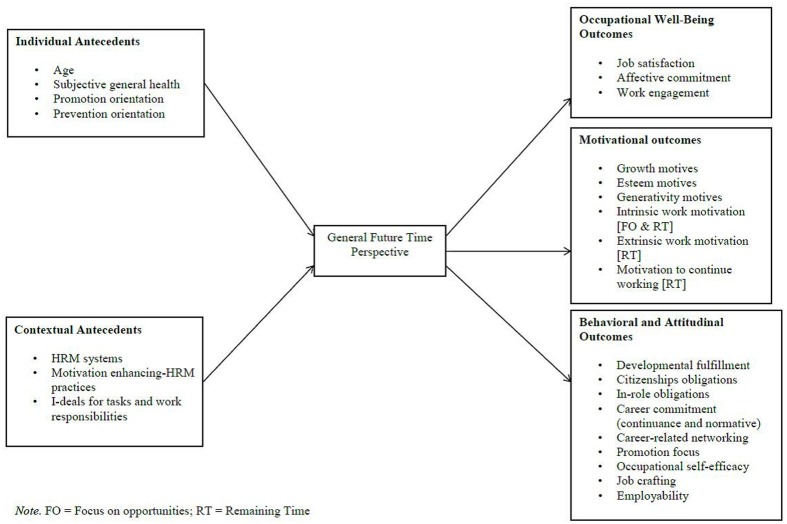
**Antecedents and consequences of general future time perspective in the work context**.

**FIGURE 3:**
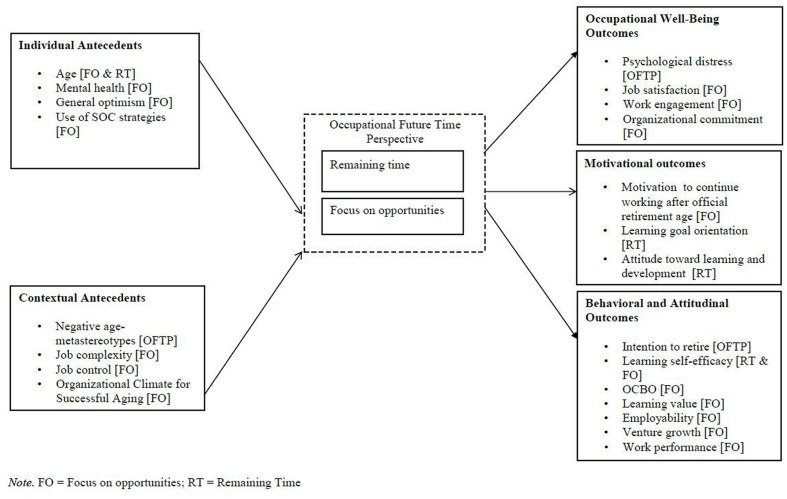
**Antecedents and consequences of occupational future time perspective**.

Since 2009, 33 published studies (see **Table 2**) have investigated the antecedents and consequences of general FTP (see **Figure 2**) and OFTP (see **Figure 3**) in the work context.

### Antecedents of Future Time Perspective at Work

#### General Future Time Perspective

Regarding individual antecedents of general FTP, age, subjective general health, and promotion focus were related to FTP, such that young, healthy workers, and those with a promotion focus had higher levels of general FTP than older and less healthy workers, and those with a prevention focus. In particular, age was negatively related to general FTP (*r* = -0.53, Bal et al., 2013), remaining time (*r* = -0.59, Kooij et al., 2014), open-ended FTP (*r* = -0.58 for sample 1 and *r* = -0.67 for sample 2; Kooij et al., 2013), and focus on opportunities (*r* = -0.44, Zacher and de Lange, 2011), and positively to limited FTP (*r* = 0.31/0.35, Kooij et al., 2013).

Subjective general health was positively related to open-ended FTP (*r* = 0.16, Kooij and Van De Voorde, 2011; *r* = 0.13/*r* = 0.17, Kooij et al., 2013) and negatively to limited FTP (*r* = -0.16; Kooij and Van De Voorde, 2011; *r* = -0.12/*r* = -0.18, Kooij et al., 2013). Moreover, Zacher and de Lange (2011) showed that a promotion focus had a positive effect on focus on opportunities (*r* = 0.47), while a prevention focus had a positive effect on focus on limitations (*r* = 0.41). Finally, for the sake of completeness, we note that sociodemographic variables, such as education and gender, have often been studied as control variables (e.g., Bal et al., 2013; Kooij et al., 2013; Yeung et al., 2013), but researchers did generally not hypothesize specific effects.

Regarding contextual antecedents of general FTP, Korff et al. (2016) reported that human resource management (HRM) systems were positively associated with general FTP (*r* = 0.24). In particular, they found relationships with motivation enhancing HRM practices (i.e., incentive compensation, internal promotion, and performance appraisal; *r* = 0.26), but not for knowledge, skills, and abilities practices neither for opportunity enhancing practices. Oostrom et al. (2016) showed that idiosyncratic deals for tasks and work responsibilities (i.e., voluntary and personalized arrangements between individual employees and their employers regarding education, tasks, or promotions) were positively related to general FTP (*r* = 0.30).

#### Occupational Future Time Perspective

As far as OFTP is concerned, younger age appears to contribute to the perception of more remaining time and opportunities left at work, and good mental health and selection, optimization, and compensation (SOC) strategies (i.e., a set of adaptive self-regulation strategies; Baltes and Baltes, 1990) are positively related to focus on opportunities. More specifically, age was strongly negatively related to remaining time at work (*r* = -0.68, Froehlich et al., 2016; *r* = -0.64, Kooij and Zacher, 2016; average *r* = -0.71, Weikamp and Göritz, 2015; *r* = -0.82, Zacher and Frese, 2009) and positively to constrained perceived remaining time (*r* = 0.81, Kochoian et al., 2016). To a lesser extent, age was negatively related to focus on opportunities at work (*r* = -0.50, Froehlich et al., 2016; *r* = -0.41, Gielnik et al., 2012; *r* = -0.48, Gielnik et al., 2016; *r* = -0.43, Kochoian et al., 2016; average *r* = -0.44, Weikamp and Göritz, 2015; *r* = -0.60, Zacher and Frese, 2009; *r* = -0.72; Zacher and Frese, 2011; *r* = -0.50, Zacher et al., 2010). Moreover, mental health (*r* = 0.20, Gielnik et al., 2012), optimism (*r* = 0.40, Schmitt et al., 2013a), and using SOC strategies (*r* = 0.09, Zacher and Frese, 2011) were positively associated with focus on opportunities.

Regarding personality, Zacher and Frese (2009) included Big Five personality traits as control variables, and found that extraversion and conscientiousness were, respectively, positively and negatively related to both focus on opportunities (*r* = 0.24, *r* = -0.28) and remaining time (*r* = 0.15, *r* = -0.22). Moreover, Zacher (2013) found positive correlations between proactive personality and focus on opportunities (*r* = 0.35) and remaining time (*r* = 0.25). Again, gender and education have often been studied as control variables (e.g., Zacher and Frese, 2009; Weikamp and Göritz, 2015). For instance, Weikamp and Göritz (2015) found effects of both gender and education, such that women and people with higher educational degrees perceived themselves as having more remaining opportunities at work. However, other studies did not find significant effects of gender (e.g., Ho and Yeung, 2016; Weikamp and Göritz, 2016).

Occupational future time perspective has also been shown to be related to contextual variables. Zacher and Frese (2009) and Zacher et al. (2010) showed that job complexity was positively associated with focus on opportunities (*r* = 0.17, *r* = 0.20). More recently, the influence of the organizational climate has also been shown. Bal et al. (2015) reported that the more older workers perceived that they were negatively stereotyped by their younger colleagues, the more their OFTP was reduced (*r* = -0.26). Conversely, Zacher and Yang (2016) found that an organizational climate for successful aging, defined as shared perceptions about organizational practices to facilitate successful aging at work, was positively associated with focus on opportunities (*r* = 0.38).

### Consequences of Future Time Perspective at Work

#### General Future Time Perspective

Regarding occupational well-being outcomes, general FTP was positively associated with job satisfaction (*r* = 0.17, Korff et al., 2016), affective organizational commitment (*r* = 0.17, Korff et al., 2016), career (*r* = 0.38) and organizational commitment (*r* = 0.34, Park and Jung, 2015), as well as physical (*r* = 0.22), emotional (*r* = 0.48), and cognitive (*r* = 0.31) engagement (Sia et al., 2015).

Regarding motivational consequences, workers with an open-ended FTP were more motivated to develop themselves at work (*r* = 0.27, Kooij and Van De Voorde, 2011; *r* = 0.32/0.22, Kooij et al., 2013) and to feel recognition, status, power, and prestige (i.e., esteem motivations) (*r* = 0.25/0.14, Kooij et al., 2013). In contrast, workers with a limited FTP were more motivated by generativity goals (*r* = 0.12, Kooij and Van De Voorde, 2011). However, regarding generativity motives, Kooij et al. (2013) did not find a significant relationship. Finally, Akkermans et al. (2016) found that both focus on opportunities and remaining time in life were positively related to intrinsic work motivations (both *r* = 0.40), extrinsic work motivations (*r* = 0.31 for focus on opportunities and *r* = 0.30 for remaining time), and motivation to continue to work (*r* = 0.27 for focus on opportunities and *r* = 0.36 for remaining time). However, when they tested their structural model, Akkermans et al. (2016) found that only focus on opportunities was related to intrinsic motivation (β = 0.32), while only remaining time was related to extrinsic motivation (β = 0.30) and motivation to continue working (β = 0.35).

For attitudinal and behavioral consequences, Bal et al. (2010) found that general FTP is positively associated with employer developmental fulfillment (i.e., workers’ perceptions that their employer has fulfilled his/her obligations regarding development, such as providing them career support and mentoring; *r* = 0.28). They did not find a significant bivariate relationship with employee obligations (i.e., resources they owe to their employer). However, when they tested a structural model, they found significant negative relationships of general FTP with both in-role obligations (e.g., quality of work and cooperation with colleagues; γ = -0.16) and citizenship obligations (e.g., being flexible about the work and working hours; γ = -0.25), but not with high performance obligations. Moreover, general FTP is positively associated with career commitment (*r* = 0.38, Park and Jung, 2015) and career-related networking (*r* = 0.22, Treadway et al., 2010). In addition, workers with high general FTP have a stronger promotion focus (*r* = 0.38, Baltes et al., 2014; *r* = 0.36, Kooij et al., 2014), higher occupational self-efficacy (*r* = 0.45, Park and Jung, 2015), and feel more employable (*r* = 0.22 for occupational expertise; *r* = 0.37 for anticipation and optimization; *r* = 0.42 for personal flexibility; *r* = 34 for corporate sense, and *r* = 0.33 for balance, Oostrom et al., 2016). Finally, Kooij et al. (2016) found that open-ended (but not limited) FTP was positively associated with job crafting behaviors (i.e., behaviors that employees engage in to improve the fit between their job and their personal needs), such as increased job resources and challenging job demands (*r* = 0.20) and decreased hindering job demands (*r* = 0.12).

General FTP has also been shown to have indirect effects through individual variables or work-related variables. For instance, general FTP has a positive indirect effect on the use of SOC strategies, through increased promotion focus (indirect effect = 0.06, Baltes et al., 2014); a negative indirect effect on turnover intention via career commitment (indirect effect = -0.25) and organizational commitment (indirect effect = -0.26; Park and Jung, 2015); and indirect positive effects on work engagement (indirect effect = 0.09) and job performance (indirect effect = 0.09) through job crafting (Kooij et al., 2016).

#### Occupational Future Time Perspective

Regarding associations of OFTP with well-being, Ho and Yeung (2016) reported a negative relationship between OFTP and psychological distress (*r* = -0.28), but a non-significant relationship with job stress. Moreover, our systematic review showed that only focus on opportunities has been investigated in relationship with well-being outcomes. Focus on opportunities was positively related to job satisfaction (*r* = 0.23, Weikamp and Göritz, 2016; *r* = 0.33, Zacher and Yang, 2016), work engagement (*r* = 0.27, Schmitt et al., 2013a; *r* = 0.31, Schmitt et al., 2013b), and organizational commitment (*r* = 0.33, Zacher and Yang, 2016).

Regarding motivational outcomes, Zacher and Yang (2016) showed that focus on opportunities was positively related to motivation to continue working after official retirement age (*r* = 0.09). Investigating perceived remaining time only, Kooij and Zacher (2016) reported positive relationships with growth motives (*r* = 0.34 for learning goal orientation; *r* = 0.45 for attitude toward learning and development).

Regarding attitudinal and behavioral outcomes, Bal et al. (2015) found that overall OFTP was associated with lower intentions to retire (*r* = -0.19). Moreover, Kochoian et al. (2016) investigated and distinguished perceived remaining time and focus opportunities. They showed that both were positively related to learning self-efficacy (*r* = 0.52 for focus on opportunities, *r* = -0.37 for constrained perceived remaining time) and learning value (*r* = 0.28 for focus on opportunities, *r* = -0.19 for constrained perceived remaining time). However, when they tested their hypotheses, they found that focus on opportunities had positive effects on both learning self-efficacy (β = 0.45) and learning value (β = 0.25), while constrained perceived remaining time had a negative effect on learning self-efficacy (β = -0.14) only. In the same way, Weikamp and Göritz (2016) as well as Froehlich et al. (2016) investigated both dimensions of OFTP and found that only focus on opportunities (and not remaining time) was positively associated with organizational citizenship behavior directed toward the organization (OCB-O; *r* = 0.29, Weikamp and Göritz, 2016) and employability (*r* = 0.30 for anticipation and optimization, *r* = 0.34 for personal flexibility, Froehlich et al., 2016). Finally, studies that investigated only the dimension focus on opportunities found positive relationships with work performance (*r* = 0.19, Zacher et al., 2010) and venture growth (i.e., changes in sales, profit, transaction volume, income, and number of employees; *r* = 0.33, Gielnik et al., 2012).

### Future Time Perspective at Work as a Mediator

#### General Future Time Perspective

Most studies have investigated general FTP as a mediator in relationships between age and work motives. For instance, Kooij et al. (2013) found that the negative relationships of age with both growth and esteem motives were mediated by an open-ended FTP, suggesting that these types of motives decrease with age because of an age-related decrease in open-ended FTP. However, they did not find that a limited FTP mediated the positive relationship between age and generativity motives. Thus, generativity motives increased with age but not with limited FTP. In a subsequent study, Kooij et al. (2014) found that perceived remaining time mediated the negative relationship between age and promotion focus.

Moreover, a few studies showed that general FTP mediated relationships between job characteristics and work-related outcomes. Oostrom et al. (2016) found that FTP mediated the positive relationships between idiosyncratic deals for tasks and work responsibilities, and employability in a sample of older workers. Korff et al. (2016) found that motivation enhancing HRM practices within the organization foster employees’ FTP, which in turn heightens affective organizational commitment.

#### Occupational Future Time Perspective

Occupational future time perspective has also been shown to act as a mediator in relationships between age and work outcomes. Studies that distinguished between perceived remaining time and focus on opportunities found that only focus on opportunities mediated the negative relationship between age and employability (Froehlich et al., 2016), and between age and learning value (Kochoian et al., 2016). In other words, older workers perceive less remaining opportunities at work and, consequently, they perceive themselves as less employable and they consider learning and development activities at work as less valuable. In addition, Kochoian et al. (2016) found that both remaining time and focus on opportunities mediated the negative relationship between age and learning-self efficacy. Investigating only perceived remaining time, Kooij and Zacher (2016) found that it mediated the negative effects of age on learning goal orientation and on attitude toward learning and development. Zacher et al. (2010) and Gielnik et al. (2012, 2016) investigated only focus on opportunities. Gielnik et al. (2012) found that it mediated the negative relationship between business owners age and venture growth. Using growth modeling analyses, Gielnik et al. (2016) found that focus on opportunities mediated the moderating effect of small business managers’ age on the relationship between time and business performance. Finally, Zacher et al. (2010) found that focus on opportunities mediated the negative relationships between age and work performance.

Similar to research on general FTP, OFTP also has been shown to mediate relationships between job characteristics and work outcomes. Zacher et al. (2010) found that focus on opportunities mediated the positive relationship between job complexity and work performance, such that employees in high-complexity jobs performed better because they had a higher focus on opportunities at work. Moreover, Bal et al. (2015) found that overall OFTP mediated the positive relationship between negative age meta-stereotypes and intention to retire, such that workers who had internalized negative age stereotypes had a lower OFTP and consequently, stronger intentions to retire.

### Future Time Perspective at Work as a Moderator

#### General Future Time Perspective

Future time perspective has been shown to moderate the employer-employee relationship. For instance, Bal et al. (2010) and De Lange et al. (2011) found that FTP moderated the relations between psychological contract fulfillment and employee obligations. In particular, Bal et al. (2010) found that the relations of economic and socio-emotional fulfillment (i.e., when employees believed that their employers has fulfilled their obligations regarding economic and socioemotional needs) with employee obligations (i.e., in-role obligations, citizenship obligations, and high performance obligations) were stronger among post-retired workers with high FTP than among post-retired workers with low FTP (Bal et al., 2010). In other words, workers with an open-ended FTP reacted more strongly to psychological contract fulfillment in relation to employee obligations, which suggest that the level of felt obligations among low FTP workers is less dependent on how they perceive employer obligations to be fulfilled (Bal et al., 2010). Similarly, De Lange et al. (2011) showed that the negative relationship between relational contract breach and work motivation was stronger among workers with a high FTP, suggesting that workers with a high FTP are more strongly affected by the way that employers behave toward them. However, a high FTP may also be a buffering resource that prevents high FTP workers against the negative impact of job stressors, such as perceived gender discrimination. In particular, Sia et al. (2015) found in a sample of female middle-aged employees that the negative relationships between perceived gender discrimination and emotional and cognitive work engagement become weaker when FTP was high.

Other studies suggest that the moderating effect of FTP depends on the independent and dependent variables under investigation. More precisely, it seems that workers tend to behave according to the needs that are most important for them, that is, socioemotional needs when FTP is low, and instrumental needs when FTP is high. For instance, Treadway et al. (2010) found that politically skilled individuals (i.e., individuals who are effective in the development, maintenance and recognition of social network) with a high FTP engaged more in career-related networking behaviors (e.g., to give business contacts a phone call to stay in touch) than politically skilled individuals with a low FTP. Moreover, Treadway et al. (2011) found that when work interfered with family, workers with a low FTP experienced lower continuance commitment, while those with a high FTP reacted to family interference with work by decreasing their level of affective commitment. Bal et al. (2013) found that socioemotional fulfillment contributes to higher continuance commitment only for low FTP workers, while high FTP workers had higher normative commitment when they received socioemotional fulfillment. Finally, Yeung et al. (2013) investigated effects of social work-related values (values related to affiliation and collaboration with coworkers) on job performance through job satisfaction, and found that the effects of these values were stronger positive among employees with low FTP. As a whole, these results are congruent with assumptions of socioemotional selectivity theory.

#### Occupational Future Time Perspective

Results of our systematic review showed that workers’ behaviors are associated with their OFTP and the congruent most important needs. Investigating coping behaviors, Ho and Yeung (2016) found that relative to those with limited OFTP, who preferred passive coping strategies, those with an open-ended OFTP preferred problem-focused and proactive coping strategies. Moreover, they found that OFTP moderated the effect of problem-focused strategies on psychological distress, such that problem-focused strategies reduced psychological distress only among workers with an open-ended OFTP. When they investigated effects of “organizational FTP” (i.e., perceived remaining time and opportunities left in the current organization), Treadway et al. (2010) found that politically skilled individuals with a limited organizational FTP were more involved in community-based networking (e.g., to attend meetings of civic and social groups, clubs and so forth) than their counterparts with an open-ended organizational FTP.

Similar to FTP, some studies indicated that OFTP may be a personal resource for workers. For instance, Schmitt et al. (2013b) found that job control, as an external resource of the work environment, is positively related to work engagement among employees with a low focus on opportunities, and not among employees with a high focus on opportunities. Similar to results from Sia et al. (2015), these results support the notion of OFTP as a compensatory resource, since a high level of focus on opportunities compensates for low levels of job control in predicting work engagement. As far as remaining time is concerned, Zacher (2013) showed that proactive personality predicts greater job search intensity when perceived remaining time is low compared to when it is high.

Finally, personal, work, and organizational resources may buffer the negative direct effect of age on focus on opportunities and on remaining time, as well as the negative indirect effects on work outcomes. For instance, Zacher et al. (2010) found that job complexity buffers the negative relationship between age and focus on opportunities, and weakens the negative indirect effect of age on work performance. In other words, when the work context offers high levels of job complexity, older workers are better able to maintain high level of focus on opportunities, and indirectly, they perform better at work. In the same way, Zacher and Yang (2016) found that older employees in organizations with a positive organizational climate for successful aging had a higher focus on opportunities than older employees who did not work in an organization with such climate. Finally, Kooij and Zacher (2016) showed that high work centrality buffered the negative relationship between age and remaining time, as well as the negative indirect effects of age on learning goal orientation and on attitudes toward learning and development.

## Discussion

In this article, we presented a comprehensive systematic review of the quantitative-empirical literature on FTP at work. Our review highlights that FTP at work has been measured and reported in various ways: some authors measured general FTP, and others measured OFTP. Authors further operationalized FTP at work as either unidimensional or bidimensional. Various individual and contextual variables are related to both general FTP and OFTP which, in turn, are related to occupational well-being, as well as motivational, attitudinal, and behavioral outcomes. Some studies investigated FTP and OFTP as mediators in relationships between age and work outcomes, and in relationships between job characteristics and work outcomes. Other studies investigated FTP and OFTP as moderators of relationships between person/contextual characteristics and work outcomes. In the following section, we will first summarize and integrate our findings.

### Summary of Findings

Results of the systematic review showed that findings are quite similar regarding antecedents and consequences of both general FTP and OFTP. As a whole, more research has investigated the work-related outcomes associated with general FTP and with the dimension focus on opportunities of OFTP. In the following sections, we will summarize results about FTP at work, and we will outline when differences were observed between general FTP and OFTP.

#### Antecedents of FTP at Work

With regard to individual antecedents, studies showed that FTP at work is associated with age, subjective health, optimism, and regulatory focus. Among contextual antecedents, our systematic review showed that both organizational characteristics, such as HRM systems and organizational climate for successful aging, as well as work characteristics, such as job control and job complexity, are related to FTP at work. Although age had the strongest negative relationship with FTP at work, several studies found that the relationship became weaker when workers have high personal (e.g., work centrality, Kooij and Zacher, 2016) or contextual resources (e.g., job control, Zacher and Frese, 2009).

#### Consequences of FTP at Work

Our systematic review showed that FTP at work, especially the dimension focus on opportunities, is positively associated with general and occupational well-being (e.g., work engagement). Consistently with socioemotional selectivity theory (Carstensen et al., 1999), FTP at work is in general positively related to growth and esteem motives, and negatively to generativity motives. With some differences depending on the type of measure, FTP at work is also positively related to work-related motives and motivation to continue working. Finally, FTP at work is positively related to a wide range of positive worker attitudes and behaviors, such as job crafting (Kooij et al., 2016) and lower intention to retire (Bal et al., 2015).

#### FTP at Work as a Mediator and as a Moderator

Studies that investigated FTP at work as a mediator found that it mediated the negative relationships between age and development-oriented attitudes, such as growth motives, promotion focus, and employability. These results suggest that development-oriented attitudes decline with age because of an age-related decrease in open-ended FTP. Moreover, FTP at work explained relationships between job characteristics and work outcomes, such as positive effects of idiosyncratic deals on employability, positive effects of HRM systems on affective organizational commitment, or positive effects of job complexity on work performance.

Findings from studies on FTP at work as a moderator showed that it moderated the relationships between psychological contract fulfillment and employee obligations; political skill and networking behaviors; work-family conflict and commitment; and stressful work situations and coping strategies. Consistent with socioemotional selectivity theory, workers with an open-ended FTP seem to be more concerned with instrumental goals, such as psychological contract fulfillment, career-related networking behaviors, continuance commitment, problem-focused and proactive coping strategies, while workers with a limited FTP emphasize more socio-emotional goals, such as community based-networking, affective commitment, social work-related values, and passive coping strategies.

### Theoretical Implications

Our review of the literature showed that over the past decade, several studies were conducted to understand the role of FTP in the work context. However, some important issues remain to be solved in future research. Our suggestions for future research are summarized in **Table 3**.

**Table 3:** Summary of future research suggestions regarding future time perspective at work.

Research focus	Research directions for studies in the work context
FTP at work antecedents	• Additional individual antecedents (e.g., personality, gender) • Additional contextual antecedents (e.g., task and skill variety, ageism, work-family interface)
FTP at work consequences	• Additional consequences (e.g., psychological health, socio-emotional motives, general well-being, intention to retire) • Distinguish dimensions (i.e., focus on opportunities and remaining time)
FTP at work as a mediator and moderator	• Status of FTP (e.g., personal resource?) • Role of FTP in the JD-R model (e.g., moderator and/or mediator?)
Research design	• Longitudinal designs • Experimental designs (e.g., vignette)
Measurement of FTP at work	• Measure OFTP more than general FTP • Emphasize the difference between the two concepts
Dimensions of FTP at work	• Measure both dimensions (focus on opportunities and remaining time) • Test the factorial structure of FTP at work • Investigate specific antecedents and consequences of both dimensions

#### Socioemotional Selectivity Theory

Future time perspective is a core construct in socioemotional selectivity theory (Carstensen et al., 1999). Socioemotional selectivity theory states that age-related changes in motives are due to changes in FTP, such that younger individuals focus more on instrumental and growth motives while older individuals focus more on socioemotional motives and relationships with close social partners. Results of our systematic review showed that FTP at work is indeed related to increased growth motives (e.g., Kooij and Van De Voorde, 2011; Kooij et al., 2013). Regarding socioemotional motives, conceptualized through the concept of generativity, results were less consistent. Results on the role of FTP at work as a mediator or as a moderator also confirmed that instrumental motives and attitudes decline with age because of an age-related decrease in open-ended FTP. Moreover, workers with an open-ended FTP seem to be more concerned with instrumental goals, while workers with a limited FTP emphasize more socio-emotional goals.

According to socioemotional selectivity theory, FTP is a flexible, cognitive-motivational, and age-related construct that changes over time (Zacher and Frese, 2009). Findings from experimental studies (e.g., Fung et al., 1999) showed that not only individual factors, but also contextual variables (e.g., life circumstances), may influence FTP. Our systematic review confirmed that other factors than age may influence FTP at work. However, these studies have mainly focused on subjective health and self-regulation strategies as individual antecedents. Regarding contextual antecedents, some work and organizational characteristics have been investigated. In the following section, we will summarize suggestions for future research on antecedents and consequences of FTP at work.

#### Antecedents of FTP at Work

Our systematic review showed that both general FTP and OFTP are influenced by similar individual and contextual antecedents. Therefore, we do not distinguish between future research suggestions for general FTP and OFTP.

##### Individual antecedents

We suggest that further studies investigate the effects of personal resources other than subjective health, optimism, and regulatory focus. In particular, personality variables such as extraversion, conscientiousness, and proactive personality might contribute to an extended FTP at work. For instance, when Zacher (2013) investigated the mediating effect of OFTP to explain the moderating role of age on the relationship between proactive personality and job search intensity, he found positive correlations between proactive personality and focus on opportunities and with remaining time at work. Moreover, controlling for Big Five personality traits, Zacher and Frese (2009) found that only conscientiousness was negatively related to focus on opportunities at work, while Cate and John (2007) found positive relationship between conscientiousness and general focus on opportunities. While Zacher and Frese (2009) explained these results by the fact that conscientious employees may focus more strongly on their present tasks and duties, Cate and John (2007) argue that conscientiousness help individuals to plan and take advantage of future opportunities. These contradictory findings highlight that future studies should take into account potential context effects to better understand the associations between personality and FTP.

The role of gender for FTP at work also requires further investigation. While some studies found gender differences in OFTP (e.g., Zacher and Frese, 2009; Treadway et al., 2010; Bal et al., 2015; Weikamp and Göritz, 2015), such that women seem to have a stronger focus on opportunities and to perceive more remaining time at work, other studies did not find significant effects (e.g., Ho and Yeung, 2016; Weikamp and Göritz, 2016). The fact that women seem to perceive themselves as having more remaining time and opportunities at work is somewhat surprising. According to Weikamp and Göritz (2015), due to the glass ceiling effect, women should perceive fewer opportunities at work than men. Moreover, the fact that they have to disrupt their work schedule because of parental leave could also lead them to perceive less remaining time at work (Weikamp and Göritz, 2015). Weikamp and Göritz (2015) suggested that this unexpected pattern may be due to their sample being composed of more educated women than the general population, which might have resulted in greater focus on opportunities. Future studies should therefore control for education when investigating gender differences in FTP at work.

##### Contextual antecedents

Work characteristics that are perceived as situational resources have been shown to extend employees’ FTP at work (e.g., Zacher et al., 2010). Future studies should investigate effects of other job resources that are particularly important for older workers. For instance, Zaniboni et al. (2013) found that increased task variety had stronger negative effects on burnout and turnover intentions among younger workers compared to older workers, while increased skill variety led to lower turnover intentions among older workers than younger workers. These results were consistent with predictions of socioemotional selectivity theory. On the one hand, task variety is likely to increase work-related knowledge that is important for future career development, which is more important for younger workers. On the other hand, skill variety will allow increasing work-related emotional-regulation goals, and increasing gratifying experiences in the present, which is most important for older workers (Zaniboni et al., 2013). On this basis, future studies could examine if positive associations of task variety and skill variety with burnout and turnover intentions are moderated by FTP at work, and if FTP explains the moderating role of age on these associations.

Moreover, it would also be interesting to analyze variables that are likely to reduce FTP at work, such as ageism. As reported by Bal et al. (2015), negative age meta-stereotypes were associated with fewer perceived opportunities until retirement. Unexpectedly, the relations were stronger among workers with a low self-categorization as an older person. These results suggest that negative stereotypes constitute a threat to workers’ self-image, especially among those who strive to maintain a positive self-image. In turn, workers might adapt by perceiving their occupational future as more limited (Bal et al., 2015). As suggested by Bal et al. (2015), more research is needed to further validate these ideas, and to investigate if the affective (i.e., prejudice) and the behavioral consequences (i.e., discrimination) of stereotypes have similar effects on FTP at work.

Finally, contextual antecedents of FTP at work related to the work-family interface have not been studied so far. However, the work-family interface may influence perceptions of remaining time at work. For instance, Raymo and Sweeney (2006) found that work-family conflict was positively related to preferences for retirement. Moreover, changes in motives depicted by socioemotional selectivity theory suggest that individuals are likely to place more importance on family relative to work when they grow older (Thrasher et al., 2015). In a study on the moderating role of general FTP in the relationship between work-family conflict and organizational commitment, Treadway et al. (2011) found negative correlations between general FTP and both work-family conflict and family work conflict. Therefore, we recommend that future studies investigate whether work-family conflict may affect perceptions of remaining time and focus on opportunities at work. Moreover, future research could investigate if the positive side of the work-family interface, such as work-family enrichment, is positively related to FTP at work. To the extent that work-family enrichment generates resources which help workers to manage work and family life (Mauno et al., 2015; McNall et al., 2015), and lead them to remain within the company (Balmforth and Gardner, 2006; Wayne et al., 2006; McNall et al., 2015), high levels of work-family enrichment might be associated with increased FTP at work.

#### Consequences of Future Time Perspective at Work

Our systematic review showed that both general FTP and OFTP have positive consequences on occupational well-being, motivation, and behavior at work. Regarding OFTP, more research investigated the consequences associated with focus on opportunities. In the following sections, we will outline our suggestions for future research on the consequences of the specific dimensions of FTP at work.

##### Occupational well-being outcomes

With the exception of a study by Ho and Yeung (2016) that reported a negative relationship between OFTP and psychological distress as measured with the General Health Questionnaire (Goldberg, 1972), we did not find studies that investigated consequences of FTP at work for general health. Instead, research on general FTP and OFTP has focused on consequences for workers’ attitudes and occupational well-being, such as affective commitment or work engagement. However, it would be interesting to investigate whether both dimensions of FTP at work are related to psychological health, since both dimensions of general FTP seem to have unique associations with health outcomes. For instance, Kozik et al. (2015) found that a high focus on opportunities was associated with less depressive symptoms and higher morale, while a low focus on limitations was associated with fewer hair cortisol. Future research should further explore if focus on opportunities and remaining time are differently related to psychological health. In particular, the expanded job demands-resources (JD-R) model by Xanthopoulou et al. (2007), which takes into account the role played by personal resources may be a suitable theoretical framework. From this perspective, it might be interesting to investigate whether focus on opportunities is a personal resource that is positively related to a positive psychological state of mind, such as work engagement, while constrained perceived remaining time (i.e., focus on limitations) is a demand that is negatively related to health, such as increased burnout.

##### Motivational outcomes

Consistent with socioemotional selectivity theory, FTP at work is related to increased growth motives, and mediated the negative effect of age on growth motives (e.g., Kooij and Van De Voorde, 2011; Kooij et al., 2013). Kooij and Van De Voorde (2011) and Kooij et al. (2013) conceptualized socioemotional motives as generativity, which refers to the concern of adults to nurture and guide younger generations (Erikson, 1963; McAdams and de St. Aubin, 1992). In the workplace, the generativity motive is defined as the preference for job features that pertain to teaching, training, and sharing skills with younger generations (Kooij et al., 2013). Results on the relationship between FTP at work and generativity motives were less consistent. While Kooij and Van De Voorde (2011) found positive relationships between limited FTP and generativity motives, Kooij et al. (2013) did not find significant relationships. It might be that generativity motives are not the best way to conceptualize socioemotional motives, especially in the work context. Future studies should use other measures of socioemotional motives, such as Yeung et al. (2013) who measured social work-related values that assess the perceived importance for social interactions and harmonious relationships with colleagues in the workplace. As shown by these authors, the positive effects of social work-related values on job performance were moderated by FTP at work, such that effects were stronger positive among employees with limited general FTP. Future studies could go further and investigate if a limited FTP at work predicts increased socioemotional motives.

Moreover, as older workers seem to experience increased level of emotional well-being (Scheibe et al., 2016), it would be interesting to test if a limited FTP explains increased well-being at the end of people’s careers. However, this proposition may raise some conceptual concerns, since research has previously shown that increased OFTP is positively associated with well-being outcomes, such as less psychological distress (Ho and Yeung, 2016) or increased job satisfaction (Weikamp and Göritz, 2015). In other words, an open-ended FTP at work is positively associated with positive well-being outcomes. Therefore, assuming that a limited FTP at work is positively associated with well-being outcomes would contradict previous findings. However, while Ho and Yeung (2016) treated OFTP as one-dimension scale and investigated general well-being, Weikamp and Göritz (2015) distinguished between both dimensions and investigated effects on work-related well-being. They found that only focus on opportunities was related to job satisfaction. On this basis, we suggest that future studies distinguish between remaining time and focus on opportunities, and investigate effects on general well-being too. In particular, limited remaining time could be positively related to general well-being rather than to work-related well-being, such as job satisfaction. As an example, couples who live in a satisfactory marriage are more prone to retire early than couples in conflict-laden marriages (Kubicek et al., 2010). In other words, high quality of relationships with family might lead people to perceive remaining time at work as limited. This perception, in turn, may be related to workers’ positive general well-being.

##### Behavioral and attitudinal outcomes

Our systematic review showed that fewer studies have investigated the behavioral consequences associated specifically with remaining time at work. Despite the fact that focus on opportunities seem to be more strongly associated with work-related variables than remaining time at work (Froehlich et al., 2016), it may be that extended perceptions of remaining time at work are related to variables such as intention to remain within the organization or intention to retire. For instance, Bal et al. (2015) found that higher global OFTP was related to lower intention to retire. Moreover, Akkermans et al. (2016) recently found that remaining time in life (but not focus on opportunities) was positively related to motivation to continue working. The fact that Bal et al. (2015) found significant effects for the global measure of OFTP while Akkermans et al. (2016) found significant effects for general remaining time only may be explained by what was measured when they referred to time. While Bal et al. (2015) measured OFTP until retirement, Akkermans et al. (2016) measured remaining opportunities and remaining time in life in general. These results suggest that intention to remain within the organization would be influenced only by the dimension remaining time. Since Bal et al. (2015) treated OFTP as a unidimensional variable, it should be interesting to replicate their study by distinguishing the two dimensions of OFTP, and to explore if, similarly, intention to retire is more strongly predicted by perceived remaining time. In a similar vein, future studies could investigate whether focus on opportunities has an indirect effect on intention to remain through improved attitudes at work (such as job satisfaction), while remaining time might have direct effect on attitudes. To date, there are not enough studies to draw definite conclusions regarding this question.

#### Future Time Perspective at Work as a Mediator and Moderator

So far, the lion’s share of studies has investigated FTP at work as a moderator or as a mediator in the relationships between age, job characteristics, and work outcomes. On the one hand, some studies found that FTP at work buffers negative associations of job demands or of a lack of job resources with work engagement. Indeed, FTP at work has been shown to be a compensatory resource that can be useful when workers face high job demands (e.g., gender discrimination, Sia et al., 2015) or a lack of job resources (e.g., job control, Schmitt et al., 2013b). On the other hand, FTP at work has been shown to mediate associations between positive job characteristics and work outcomes. For instance, Korff et al. (2016) showed that FTP at work mediated the positive relationship between motivation-enhancing HRM practices and affective organizational commitment.

Results from these studies suggest that FTP at work constitutes a personal resource that can either moderate or mediate positive relationships between job characteristics and work outcomes. However, the simultaneous presence of both moderation and mediation hypotheses and associated empirical findings in the literature may raise the question whether FTP at work plays a systematic role in the relationships between job characteristics and work outcomes. Moreover, there appears to be a lack of a theory to argue why FTP at work acts either as a mediator or as a moderator in these relationships.

In our view, this issue may be related to the various roles of personal resources in the JD-R model (Xanthopoulou et al., 2007). As highlighted by Schaufeli and Taris (2014), personal resources (i.e., the psychological characteristics that are generally associated with resiliency and that refer to the ability to control and impact one’s environment successfully) may play at least five different roles in the JD-R model. For instance, some studies found that personal resources moderate the relationships between job characteristics and well-being outcomes (e.g., Van den Broeck et al., 2011), while others found that personal resources mediate this relationship (e.g., Xanthopoulou et al., 2007).

To clarify the role played by FTP at work, future studies should systematically test and compare different conceptualizations of the relationships between job characteristics, FTP at work, and work outcomes. Moreover, studies that investigated FTP at work either as a mediator or as a moderator should refer to a strong theoretical background to support their hypotheses. To this end, the expanded Job Demands-Resources model by Xanthopoulou et al. (2007), which takes the role of personal resources (i.e., self-efficacy, organizational-based self-esteem, and optimism) into account, as well as conservation of resources theory (Hobfoll, 1989), which suggests that employees working in a resourceful environment will become more confident and optimistic about their future at work, may be informative.

Future research could also integrate OFTP with general theories of work and aging, such as the action regulation across the adult lifespan (ARAL) framework (Zacher et al., 2016). Based on action regulation theory, the ARAL framework suggests that workers regulate their actions by developing and selecting goals, orienting themselves in the environment, planning, monitoring the execution of behavior, and processing feedback. Zacher et al. (2016) argued that aging and age-related changes in person and contextual factors impact on this action regulation process. Change in OFTP might be an important mediating mechanism in this regard. For instance, OFTP might influence whether young, middle-aged, and older workers set short- or long-term goals (Seijts, 1998), and what kind of information workers prioritize when processing external feedback (Wang et al., 2015). Integrating OFTP with the ARAL framework appears to be an important step toward an improved, theory-based understanding of how work behavior changes across the working life span.

#### Research Design

Regarding the research designs of articles included in our review, we note that the majority of studies were cross-sectional. Future studies should make use of longitudinal designs more often to test the causal direction of relationships, and to assess the dynamics of FTP at work. Results from a longitudinal study with six measurement waves over 4 years showed that OFTP decreased over time, and that the rate of decrease in OFTP was associated with age (Weikamp and Göritz, 2015). In particular, the study found that workers perceived fewer remaining time and opportunities over time, and younger workers felt that their remaining time decreased faster than older workers did. Thus, the relationship between age and FTP at work might not be linear and future studies should investigate further how FTP at work decreases over time and depending on age. Furthermore, as suggested by Weikamp and Göritz (2015), future studies could investigate whether FTP as conceptualized by Cate and John (2007), which distinguishes between focus on opportunities and focus on limitations, decreases also depending on age. Finally, a longitudinal design would also be useful to assess whether effects of both dimensions of FTP at work on work outcomes are always linear and positive, or whether they might also be curvilinear under certain circumstances. For instance, future research could investigate whether perceiving a lot of remaining time at work has positive effects when workers are not satisfied at work.

In addition, future studies could induce a limited vs. an open-ended OFTP in experiments or through situational vignettes. Such designs have already been used in studies assessing effects of open-ended vs. limited general FTP on preferences for social partners (e.g., Fung et al., 1999; Fung and Carstensen, 2004). To apply this to the work context, future studies could, for instance, manipulate the official age for retirement through a vignette, and see if this affects workers’ perceived remaining time and focus on opportunities at work.

#### Measurement of Future Time Perspective at Work

A major issue regarding current research on FTP at work concerns the use of different measurement instruments. Some researchers have either measured general FTP, which involves remaining time and opportunities in life in general, while others measured OFTP, which entails remaining time and opportunities at work. Perceptions of remaining time *in life* (i.e., general FTP) make salient the subjective life expectancy, whereas perceptions of remaining time *to work* (i.e., OFTP) make salient the expected age for retirement. Moreover, the items of the OFTP scale focus on the work sphere, whereas general FTP has a more global focus. For instance, general FTP may refer to time left and opportunities in the work sphere but also in the private sphere. Furthermore, using a measure of general FTP or OFTP may lead to different results. For instance, Treadway et al. (2010) measured general FTP (i.e., perceptions of time left in life in general) and OFTP (i.e., perceptions of time left in a specific organization). Results showed that FTP, but not OFTP, was related to career and community networking behaviors, such as giving business contacts a phone call to stay in touch or attending meetings of civic and social groups.

Finally, our systematic review showed that most studies conducted in the workplace did not clearly specify whether they measured general FTP or OFTP. To be less ambiguous, we suggest that future studies conducted in the work context use the OFTP scale, because it has been specifically adapted to the work context. If researchers are interested in investigating effects of general FTP in worker samples, we recommend that they explain why they measure general FTP instead of OFTP, and describe the difference between the two constructs (e.g., see Akkermans et al., 2016).

#### Dimensions of Future Time Perspective at Work

Another issue concerns the structure of FTP at work. The original scale developed by Carstensen and Lang (1996) was initially conceived as unidimensional, but contains items assessing both remaining time and focus on opportunities. While several researchers (e.g., Cate and John, 2007; Zacher and Frese, 2009) found that FTP at work is best described by two dimensions, most studies on FTP at work implicitly assume that the FTP scale is unidimensional, but they do not test this assumption. However, some authors who assessed the structure of general FTP found that a two-factor model, with remaining time and focus on opportunities, fitted the data better than a one-factor model (e.g., Kooij et al., 2014). On this basis, we recommend that researchers systematically test whether a two-factor model of FTP at work fits the data better than a one-factor model.

Moreover, future studies should systematically test whether focus on opportunities and remaining time have different antecedents and consequences. Some studies suggest that both dimensions may differ with regard to the relationships they have with individual and organizational variables. Regarding its antecedents, focus on opportunities seems to be more strongly related to working conditions than remaining time. For instance, Zacher and Frese (2009) found that focus on opportunities (but not remaining time) was associated with work characteristics (i.e., job control and job complexity). As mentioned by Weikamp and Göritz (2015), people who change their job or team, or get a new supervisor, will probably perceive themselves as having more or less opportunities after the change. On the one hand, these kinds of work-related changes seem less likely to influence perceptions of remaining time at work. On the other hand, perceptions of remaining time at work are more strongly related to age than focus on opportunities, probably because most people retire within a narrowly defined age range.

Regarding the consequences, Weikamp and Göritz (2016) found that focus on opportunities (but not remaining time) has positive relationships with job satisfaction and organizational citizenship behavior. Moreover, Froehlich et al. (2016) as well as Kochoian et al. (2016) found that only focus on opportunities (but not remaining time) was positively associated with employability and learning value. These results suggest that focus on opportunities is more directly related to work outcomes than remaining time. This may explain why we found more studies that investigated only the concept focus on opportunities and its relationships with work outcomes. Today, there are not enough studies to develop differentiated hypotheses for focus on opportunities *and* remaining time. We thus recommend that researchers include both dimensions in their studies and investigate whether they have the same relationships with individual and contextual antecedents.

Interestingly, some results of mediation analyses suggest that remaining time may be an antecedent of focus on opportunities. For instance, Kooij et al. (2014) reported that perceived remaining time mediated the negative relationship between age and promotion focus, which is related to focus on opportunities (Zacher and de Lange, 2011). Future studies could investigate the lagged relationships between both dimensions, and test whether remaining time is an antecedent of focus on opportunities, and whether they interact to predict work outcomes.

### Practical Implications

Our review showed that an increased FTP at work is associated with positive consequences for individuals (e.g., less psychological distress, increased employability) and organizations (e.g., lower intention to retire, increased work performance). Since perceptions of remaining time and focus on opportunities are positively associated with contextual variables, such as positive work characteristics, organizations should aim to improve these job characteristics. For instance, managers could be trained to redesign jobs to allow for more autonomy (i.e., job control) and more challenging tasks (i.e., job complexity). To enhance FTP at work among older workers, practitioners could change job characteristics that are particularly important for this group of workers. For instance, managers could increase skill variety to allow older workers to make full use of their experience-based knowledge (Truxillo et al., 2012).

Organizational climate may also influence perceptions of remaining time and focus on opportunities at work. To decrease negative age stereotypes, training on how to manage age-diversity could be provided to managers. As an example, Ries et al. (2013) designed and implemented such training for managers. Results showed that the training had a positive impact 4 months later, by increasing appreciation of age diversity, and by reducing age stereotypes of supervisors (Ries et al., 2013). In addition, other interventions could be implemented to create an organizational climate that supports all age groups, and to reduce negative age stereotypes. For instance, intergenerational contact has been shown to be negatively related to ageism and turnover intentions (Iweins et al., 2013; Henry et al., 2015). Moreover, applying age-inclusive HR practices (e.g., age-neutral recruiting activities, equal access to training for all age groups) can positively influence perceptions of an organization-wide age diversity climate (Böhm et al., 2014).

Finally, the use of SOC strategies has been shown to be positively related to focus on opportunities (Zacher and Frese, 2011; Baltes et al., 2014). Therefore, SOC training could be provided to employees to teach them how to better select their work goals, optimize goal pursuit, and compensate for the loss in relevant resources (Moghimi et al., 2016). To this end, the SOC training developed by Müller et al. (2015) may be an important tool for organizations. During the training, each participant choses a specific goal, develops an action plan to achieve this goal in an optimal way, and considers alternative strategies to face the possible hindrances during goal pursuit. The goals have to be either to cope more effectively with an important job demand, or to activate a valued job resource. Applying this training among nurses working in a community hospital, Müller et al. (2015) observed a trend that the proposed SOC training increased mental well-being, especially among nurses who were strongly committed to the intervention. Since the use of SOC strategies is particularly beneficial for maintaining older workers’ focus on opportunities (Zacher and Frese, 2011), especially when they have a low-complex job, organizations should provide them with training on the use of SOC strategies.

## Key Contributions and Conclusion

In our systematic review, we summarized quantitative-empirical studies on FTP at work. Despite a growing number of studies conducted on this topic, there are still many opportunities for future research. With regard to conceptual issues, for example, researchers could investigate additional antecedents (e.g., gender, personality, work-family interface) and further consequences of FTP at work (e.g., psychological health, socioemotional motives, retirement outcomes). Moreover, we identified several methodological issues and areas for future research (e.g., dimensionality of scales, longitudinal and experimental designs).

More specifically, our systematic review contributes to the literature by identifying four important areas for future research. First, more research is needed regarding the role of FTP at work for explaining age differences in emotional functioning and well-being. As highlighted in the review, results regarding relationships of FTP at work with socioemotional motives (e.g., generativity) were not consistent. Moreover, the use of FTP as an explanatory variable for observed age differences in emotional functioning has recently been questioned (Grühn et al., 2016). Future research could investigate whether a limited FTP at work, consistent with predictions of socioemotional selectivity theory, is positively associated with subjective well-being among older workers, and whether FTP at work acts as an explanatory mechanism.

Second, the role of FTP at work for relationships between work characteristics and work outcomes should be clarified. Our review identified many studies that investigated FTP at work as either a mediator or as a moderator in these relationships. Future research should test and compare different conceptualizations of the relations among job characteristics, FTP at work, and work outcomes. A strong theoretical background is also needed to determine how FTP at work should be integrated in such models.

Third, our review identified a lack of consistency regarding the way FTP at work is measured and analyzed. There is a need for systematic research that simultaneously tests and compares relationships of general FTP and OFTP with antecedents and outcomes. The results could be informative as to whether the general items by Carstensen and Lang (1996) or the adapted items by Zacher and Frese (2009) lead to the same conclusions. Moreover, further research is needed regarding the consequences of conceptualizing FTP at work as having one, two, or even three dimensions (i.e., including a focus on limitations; Zacher, 2013).

Fourth and finally, the majority of studies we reviewed were based on self-reports at a single point in time and non-experimental designs, and thus do not allow drawing conclusions about causality. Since FTP at work has been shown to decrease over time (Weikamp and Göritz, 2015), it is crucial that the research design of future studies takes into account the role of time. Another potential study opportunity is to examine whether, similarly to general FTP in non-work specific samples, FTP at work can be manipulated in samples of workers.

In summary, the results of our systematic review showed that both general FTP and OFTP are associated with various individual and contextual antecedents, and that extended perceptions of remaining time and focus on opportunities are, in general, associated with positive individual and work-related outcomes. Thus, our findings suggest that individual workers and organizations may benefit from extended perceptions of remaining time and focus on opportunities at work.

## Author Contributions

HH: substantial contributions to the conception or design of the work; drafting the work; final approval of the version to be published; agreement to be accountable for all aspects of the work in ensuring that questions related to the accuracy or integrity of any part of the work are appropriately investigated and resolved. HZ, DD: substantial contributions to the conception or design of the work; revising it critically for important intellectual content; final approval of the version to be published; agreement to be accountable for all aspects of the work in ensuring that questions related to the accuracy or integrity of any part of the work are appropriately investigated and resolved.

## Conflict of Interest Statement

The authors declare that the research was conducted in the absence of any commercial or financial relationships that could be construed as a potential conflict of interest.
